# Genotype identification of *Enterocytozoon bieneusi* isolates from stool samples of HIV-infected Tunisian patients

**DOI:** 10.1051/parasite/2012192147

**Published:** 2012-05-15

**Authors:** N. Chabchoub, R. Abdelmalek, J. Breton, F. Kanoun, M. Thellier, A. Bouratbine, K. Aoun

**Affiliations:** 1 Laboratoire de recherche “Parasitoses médicales, biotechnologie et bio-molécules” LR 11-IPT-06 & Laboratoire de parasitologie, Institut Pasteur de Tunis Tunisie; 2 Service des maladies infectieuses, Hôpital de la Rabta Tunis Tunisie; 3 Hôpital de la Pitié-Salpétrière, Unité INSERM 511, Université Paris VI Pierre et Marie Curie Paris France

**Keywords:** microsporidia, *Enterocytozoon bieneusi*, genotype, Internal Transcribed Spacer, HIV, Tunisia, microsporidie, *Enterocytozoon bieneusi*, génotype, ITS, VIH, Tunisie

## Abstract

The microsporidian species *Enterocytozoon bieneusi* is a major cause of chronic diarrhea and malabsorption in patients with AIDS. Genotyping was performed on seven *E. bieneusi* strains for the first time in Tunisia. All the strains were isolated from stool samples of humans with immunodeficiency virus (HIV) infection. Analysis of the ribosomal RNA gene internal transcribed spacer (rDNA ITS) allowed the identification of three distinct genotypes previously described in other studies. Genotypes D and B were characterized in four and two respectively. The Peruvian genotype (Peru 8) was detected in the last isolate. These results indicate a genetic diversity in *E. bieneusi* strains from HIV Tunisian patients and suggest the coexistence of both zoonotic and anthroponotic route of transmission.

## Abstract (fr)

*Enterocytozoon bieneusi* est l’espèce de microsporidies la plus fréquente chez l’homme. C’est l’une des causes majeures de diarrhées chroniques et de malabsorption chez les patients atteints de SIDA. Le génotypage de souches d’*E. bieneusi* a été effectué pour la première fois en Tunisie. Il a concerné, par l’analyse de la séquence ITS du gène codant l’ARN ribosomique (rDNA ITS), sept isolats obtenus de selles de sujets infectés par le virus de l’immunodéficience humaine (VIH). Trois génotypes différents ont été identifiés. Le génotype D et le génotype B ont été détectés respectivement dans quatre et deux isolats. Le génotype Peru 8, antérieurement décrit uniquement au Pérou, a été détecté dans le dernier isolat. Ces résultats témoignent de la diversité génétique des souches d’*E. bieneusi* en Tunisie et suggèrent la coexistence de modes de transmission aussi bien zoonotiques qu’anthroponotiques.

## Introduction

Microsporidia are obligate intracellular pathogens that emerged in humans during AIDS epidemic. The most identified Microsporidia species is *Enterocytozoon bieneusi* (Didier, 2005). It is often associated with chronic diarrhea and wasting in immunocompromised patients especially those infected with human immunodeficiency virus (HIV) (Endeshaw *et al.*, 2006). Despite its isolation in animals and environmental samples, sources and transmission modes to human of *E. bieneusi* are still not well established (Coupe *et al.*, 2006; Mathis *et al.*, 2005). Molecular tools based on analysis of internal transcribed spacer (ITS) of rRNA gene, notably those developed by Rinder and Liguory have been employed to delineate the transmission of *E. bieneusi* (Rinder *et al.*, 1997; Liguory *et al.*, 2001). The method of Rinder based on sequencing of the 243 bp internal ITS region has highlighted a considerable genetic diversity within *E. bieneusi* isolates with more than 50 distinct genotypes reported to date in humans and animals. Some of these genotypes are host-specific and other have zoonotic potential (Thellier & Breton, 2008; Santin *et al.*, 2008). In Tunisia, the prevalence of *E. bieneusi* infection in patients with HIV was determined at 5.9% (Chabchoub *et al.*, 2009), but genotypic data are not available. The purpose of this study was to identify genotypes of *E. bieneusi* strains isolated from stool samples of HIV-infected Tunisian patients using sequence analysis of the ITS region located in rRNA genes.

## Materials and Methods

### Stool samples and clinical information

Seven *E. bieneusi* positive stool specimens from HIV-infected patients were collected during the period of 2005-2008 at the infectious diseases department of Rabta hospital, Tunis. The patients mean age was 28.9 years and the sex ratio was 0.4 (male/female). Five patients had CD4 cell counts below 50 cell/mm^3^ and four patients presented with diarrhea (Table I).

**Table I.** Clinical and epidemiological data of patients with *Enterocytozoon bieneusi* infection.

Patient	Sex	Age (years)	Habitat	Stool aspect	CD4 count (cells/μl)	ITS (Genotype)
Tn14	F	39	Urban	Molded	23	B
Tn15	F	1 month	Urban	Molded	991	B
Tn42	M	40	Urban	Diarrheal	22	Peru 8
Tn44	M	32	Urban	Molded	44	D
Tn106	F	29	Urban	Diarrheal	17	D
Tn110	F	24	Rural	Diarrheal	NA	D
Tn124	F	34	Rural	Diarrheal	4	D

F: female, M: male, NA: not available.

### Screening for microsporidian spores

All stool samples were analyzed by both light microscopy (Weber’s modified trichrome stain) and PCR using universal primers V1/PMP2 as previously described (Chabchoub *et al.*, 2009; Fedoroko *et al.*, 1995). PCR using species-specific primers V1/EB450 as previously described allowed the identification of *E. bieneusi* (Coyle *et al.*, 1996).

### DNA extraction

The DNA was extracted from frozen samples using the QIAamp DNA Tissue Kit (Qiagen Inc, Germany). Briefly, 200 μL of stool suspension were washed three times in phosphate-buffred-saline (PBS) solution by centrifugation at 12,000 g for five minutes. The pellet was then resuspended in 50 μL of cetylmethylammonium bromide at 2% (CTAB) and 180 μl of tissue lysis buffer and incubated with proteinase K during two hours at 55 °C. The manufacturer’s recommendations were followed for DNA purification and elution. Extracted products were stored at -20 °C.

### PCR and sequencing of the ITS region

PCR reactions were done using MSP3A (5’-GGAATTCACACCGCCCGTCRYTAT- 3’) and MSP4B (5’-CCAAGCTTATGCTTAAGTCCAGGGAG- 3’) primers, which amplify a 508 bp fragment containing 122 bp of the 3’end of the small-subunit rRNA gene, 243 bp of the ITS, and 143 pb of the 5’ region of the large-subunit rRNA gene (Rinder *et al.*, 1997). PCR amplification was performed in 50 μL reaction mixtures containing 1X PCR buffer, 2.5 mM of MgCl_2_, 25 pmol of each primer, 200 μM of each dNTP, 2 U of Goldstar Taq DNA polymerase (Applied biosystems, Roche, Switzerland), and 5 μL of DNA. PCR conditions were 94 °C for 2 min, followed by 40 cycles of 1 min at 94 °C, 1 min at 55 °C, and 1 min at 72 °C; with a final extension phase at 72 °C for 4 min. Amplified products were separated by electrophoresis on a 2 % agarose gel and visualized after staining by ethidium bromide. Negative (distilled water) and positive (DNA extracted from *E. bieneusi* positive sample) controls were systematically included.

PCR products were sequenced in both directions with a Big Dye Terminator Cycle sequencing kit on ABI PRISM 377 DNA sequencer (Applied Biosystem, USA). The genotype of *E. bieneusi* from each specimen was classified by comparing sequence to those available in the GenBank database (http://www.ncbi.nlm.nih.gov) and by alignments performed by Clustal X 1.83 for windows (Thompson *et al.*, 1994).

### Nucleotide sequence accession numbers

The GenBank accession numbers for 3 distinct obtained sequences Tn14, Tn106 and Tn42 are JF797332, JF797333 and JF797334, respectively.

## Results

**Fig. 1.**
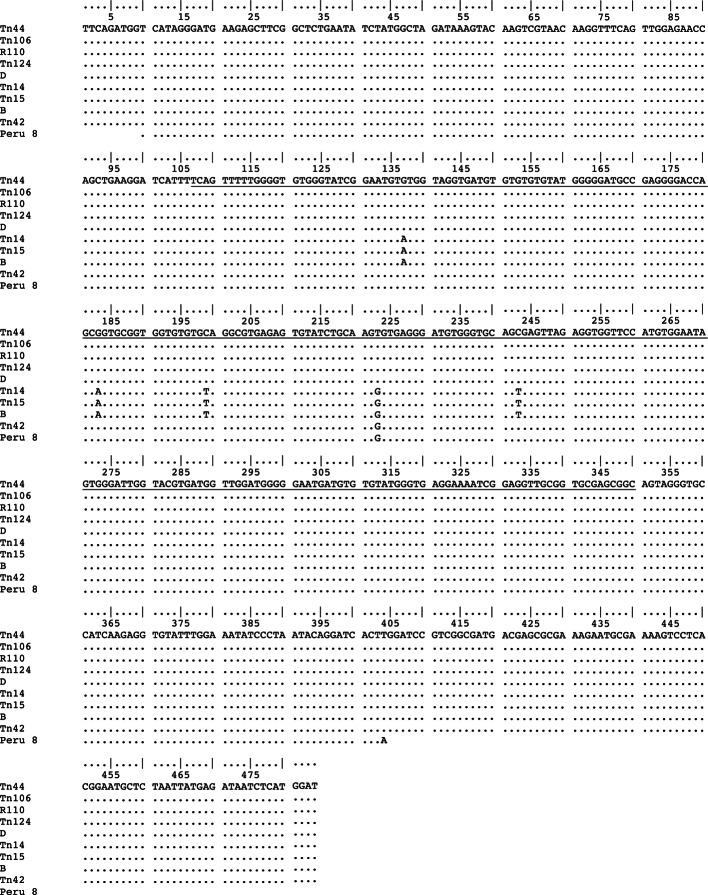
DNA sequence alignment of the rRNA genes sequences of *E. bieneusi*. Our sequences (Tn44, Tn106, Tn110, Tn124, Tn14, Tn15 and Tn42) are compared with sequences available in Genbank Genotypes B (accession no. AF101198), D (accession no. AF101200) and Peru 8 (accession no. AY371283). Dots denote sequence identity. The 243 bp of ITS region is underlined. The beginning of the sequence corresponds to the last 106 bp of small-subunit rRNA gene and the end corresponds to the first 135 bp of large-subunit rRNA gene.

PCR products of 508 bp in size were successfully amplified for the seven stool samples. Sequencing of these DNA fragments indicated that all the sequences corresponded to *E. bieneusi*. The analysis of ITS sequences of the seven isolates revealed three *E. bieneusi* genotypes previously described as B, D and Peru 8 (GenBank accession nos AF101198, AF101200, AY371283). Polymorphic sites were revealed by comparison to previously reported ITS sequences (genotypes B, D and Peru 8) as shown in Fig. 1. Four isolates (Tn44, Tn106, Tn110, Tn124) were identical to the published *E. bieneusi* ITS sequences from genotype D (also identified as genotype pigEBITS9, WL8, Peru9, coch112 and Falco5) isolated in humans and animals. Genotype B, also characterized as genotype I, was found in two isolates (Tn14, Tn15). The last sequence (Tn42) displayed identity to Peru 8 genotype.

The ITS flanking regions (122 bp of the small-subunit rRNA and 143 bp of the large-subunit rRNA) of the seven sequences showed 100% identity to the corresponding regions of *E. bieneusi* rRNA sequences from genotype B, D and Peru 8 (Fig. 1). Genotype Peru 8 is shorter (395 bp) than the others because Sulaiman *et al.* used different primers (Sulaiman *et al.*, 2003). Nucleotide A (position 404) located out 243 bp of the ITS sequence is likely an error in the GenBank sequence of Peru 8 (Sulaiman *et al.*, 2003).

The two genotype B strains were isolated from patients with molded stool living in urban area. Genotype D strains were detected in patients with both urban and rural origin and presented both molded and diarrheic aspects of stool. Genotype Peru 8 was isolated from a diarrheic patient living in an urban area (Table I).

## Discussion

The genotyping of *E. bieneusi* isolates is a valuable tool for epidemiological investigation (Breton *et al.*, 2007). The present study demonstrates that ITS sequence analysis is applicable to typing and classification of *E. bieneusi* strains isolated from HIV Tunisian patients stools. Despite its high cost and technical complexity, ITS sequencing is the most commonly used method for epidemiological studies (Breton *et al.*, 2007; Touzeau *et al.*, 2000). It has a high discrimination power and permits the identification of multiple and new genotypes (Espern *et al.*, 2007; Leelayoova *et al.*, 2006). In fact, ITS sequencing allows the detection of very subtle differences (one base pair) between sequences. To date, at least 34 different genotypes of *E. bieneusi* have been reported in humans (Thellier & Breton, 2008). Several studies indicate that the genotypes of *E. bieneusi* probably differ according to their geographical distributions (Breton *et al.*, 2007; Touzeau *et al.*, 2000; Espern *et al.*, 2007). Besides, it has been recently proposed that predominant genotypes could be related in different sites to distinct transmission sources (Breton *et al.*, 2007). The current study reports for the first time identification of *E. bieneusi* genotypes in North Africa. The results showed three distinct genotypes in *E. bieneusi* strain isolated from HIV infected Tunisian patients with intestinal microsporidiosis.

All identified genotypes were previously described elsewhere. The most frequently observed genotype D (n = 4) has a big variety of hosts and a large geographic range. It was first demonstrated in human in Germany then in American, Asian and African countries (Rinder *et al.*, 1997; Breton *et al.*, 2007; Leelayoova *et al.*, 2006; Sulaiman *et al.*, 2003). It was also observed in a wide variety of isolates from domestic (cattle and cat) and wild animals (macaque, muskrat, beaver and fox) supporting a zoonotic route of transmission (Breton *et al.*, 2007; Dengjel *et al.*, 2001). Genotype B, observed in two patients, is considered host specific and was recovered exclusively from humans (Rinder *et al.*, 1997; Liguory *et al.*, 2001 Sulaiman *et al.*, 2003; Breitenmoser *et al.*, 1999). It is the most common genotype reported in HIV-positive patients in Europe and has been recently isolated from three HIV positive patients in Cameroon (Breton *et al.*, 2007). Genotype B is also the dominant strain in France, Germany, Switzerland and United Kingdom, making up 50% to 85% of the isolates (Rinder *et al.*, 1997; Liguory *et al.*, 2001; Breitenmoser *et al.*, 1999; Sadler *et al.*, 2002). The last identified genotype Peru 8 was described only in HIV patients in Peru, where genotype D has been also reported but not genotype B (Sulaiman *et al.*, 2003). To our knowledge, this is the second report of Peru 8 genotype in the world.

According to such results, different transmission modes of *E. bieneusi* could exist in Tunisia. The predominance of genotype D in our patients (57.1%) suggests a frequent transmission from animals to humans after exposure to animals or to contaminated surface water. On the other hand, the presence of anthroponotic genotypes B and Peru 8 underlines also the person to person transmission, facilitated probably by enteric carriage of *E. bieneusi* spores as supported by previous studies (Bretagne *et al.*, 1993; Tumwine *et al.*, 2005). Further studies including larger numbers of human and animal isolates are needed to confirm both hypotheses.
